# Novel Assignment of Gene Markers to Hematological and Immune Cells Based on Single-Cell Transcriptomics

**DOI:** 10.3390/ijms26020805

**Published:** 2025-01-18

**Authors:** Enrique De La Rosa, Natalia Alonso-Moreda, Alberto Berral-González, Elena Sánchez-Luis, Oscar González-Velasco, José Manuel Sánchez-Santos, Javier De Las Rivas

**Affiliations:** 1 Cancer Research Center (CiC-IBMCC, CSIC/USAL/IBSAL), Consejo Superior de Investigaciones Científicas (CSIC), University of Salamanca (USAL) & Instituto de Investigación Biomédica de Salamanca (IBSAL), 37007 Salamanca, Spain; enriquedlrm98@usal.es (E.D.L.R.); nataliaalonsom@usal.es (N.A.-M.); aberralgonzalez@usal.es (A.B.-G.); elenasl@usal.es (E.S.-L.); jose@usal.es (J.M.S.-S.); 2 Division of Applied Bioinformatics, German Cancer Research Center (DKFZ), 69120 Heidelberg, Germany; 3 Department of Statistics, University of Salamanca (USAL), 37008 Salamanca, Spain

**Keywords:** single cell, RNA-seq, human gene, gene signature, cell marker, biomarker, CD, blood, bone marrow, immune cell, bioinformatics, machine learning

## Abstract

There are many different cells that perform highly specialized functions in the human hematological and immune systems. Due to the relevance of their activity, in this work we investigated the cell types and subtypes that form this complex system, using single-cell RNA sequencing (scRNA-seq) to dissect and assess the markers that best define each cell population. We first developed an optimized computational workflow for analyzing large scRNA-seq datasets. We then used it to find gene markers of the different cell types present in bone marrow (BM) and peripheral blood (PB). We analyzed three different single-cell datasets to find specific cell markers using this strategy: first, we searched in the CD marker genes and then in the genes encoding membrane proteins and finally in all detected protein-coding genes. This allowed us not only to confirm known CDs that best mark some cell types (e.g., monocytes, B cells, NK cells, etc.) but also to test the ability of new genes to distinguish specific cell types. Finally, we applied a machine learning method (Random Forest) to test the accuracy of the different markers we found. As a result of all this work, we have found and propose specific and robust gene signatures to identify different types and subtypes of hematological and immune cells.

## 1. Introduction

Hematopoiesis is the complex process where an enormous number of adult cells are constantly regenerated throughout life, producing multiple populations of highly specialized cells of different types with unique functions [1]. In humans, this process occurs primarily in the adult bone marrow (BM), and the hematopoietic system is divided into four major cellular lineages: erythroid lineage, megakaryocyte lineage, myeloid lineage, and lymphoid lineage [2]. The main cell types in the myeloid lineage are the following: myeloid dendritic cells, plasmacytoid dendritic cells, classical monocytes, non-classical monocytes, and neutrophils. The main cell types in the lymphoid lineage are T cells (with many subtypes, such as CD4+ T cells and CD8+ T cells), B cells, plasmatic cells, and natural killer (NK) cells. All these cells play different roles in the immune system and in the multiple physiological functions carried out by the hematological system. Therefore, the molecular characterization, isolation, and analysis of these different types of immune and hematological cells have received growing interest in biomedical studies, especially for the advancement of cell therapies and regenerative medicine and for a better characterization of many diseases that cause specific cytopenias or abnormal cell growth (as is the case in many hematological malignancies, such as leukemias, lymphomas, etc.) [3].

The different cell populations are commonly classified by clusters of differentiation (CDs), which are surface molecular markers of the cells used for immunophenotyping and distinguishing between the different cell types and subtypes [4]. These markers, once identified as characteristic of certain specific cells, are called CDs to follow a common nomenclature that allows for better identification and sharing within the international scientific and research community [5]. These surface markers have specific functions depending on the cell and can be differentially expressed when the cells undergo intracellular genetic alterations or when there are changes in cell state or changes in environmental conditions [6,7]. In humans, the current CD collection includes a list of 371 molecules (mainly proteins) designated by the Human Cell Differentiation Molecules (HCDM) council (http://www.hcdm.org/) [6,7] and reported in various biomolecular databases; for example, in UniProt (human cell differentiation molecules: CD nomenclature and list of entries; cdlist.txt). In general, CDs are well-defined and contrasted cell markers, but new markers are still needed to identify and specify many subtypes and subpopulations of human cells. An example of this is that some of the most recent CDs added to the list are C-type lectins with the identifications CD367, CD368, CD369, CD370, and CD371 [5]. These membrane markers are very relevant in the clinic, because abnormal expression of these CDs in bone marrow and peripheral blood is one of the first diagnostic features to identify patients with different types of leukemia [4]. This demonstrates the enormous importance of generating deeper and more precise knowledge about the molecular markers that can identify specific cell types or trace particular alterations in specific cells.

Transcriptomics and full gene expression profiling of many human samples from different organs, tissues, and cells over the past decades have provided a large corpus of knowledge about the protein-coding genes that are present in different cell types or at different stages of the cells in different human cell lineages. Many of these studies have been performed on the hematologic lineage because, as noted above, it encompasses many different cell types. Despite these advances, current CD markers are unable to separate some subpopulations with similar molecular profiles that are functionally distinct, such as naive and memory CD4+ T cells, or naive and memory CD8+ T cells, or regulatory T cells (Tregs) and helper T cells. In this context, technologies such as single-cell RNA sequencing (scRNA-seq) can uncover complex and unknown populations without losing cellular differences, revealing regulatory relationships between genes and showing the evolutionary relationships of different cells [8]. Because of that, in this work, we evaluate the performance of several widely used markers (CDs) and discover other genes or membrane proteins that mark specific human hematological cells.

## 2. Results

### 2.1. Single-Cell RNAseq Datasets

We collected three independent transcriptomic datasets of human samples generated by single-cell RNA sequencing (scRNA-seq), containing single-cell gene expression data from different human primary cells (see Table 1 in Section 2, Materials and Methods, for a full description of the characteristics of these datasets) [9,10,11]. The datasets were collected from a variety of published resources and generated using the 10x Genomics Chromium platform for cell isolation and the Illumina HiSeq 3000, HiSeq 4000, and NovaSeq 6000 high-throughput sequencing platforms for transcriptomic expression profiling.

### 2.2. Single-Cell Analysis Workflow and Identification of Hematological and Immune Cell Types

**Figure 1:**
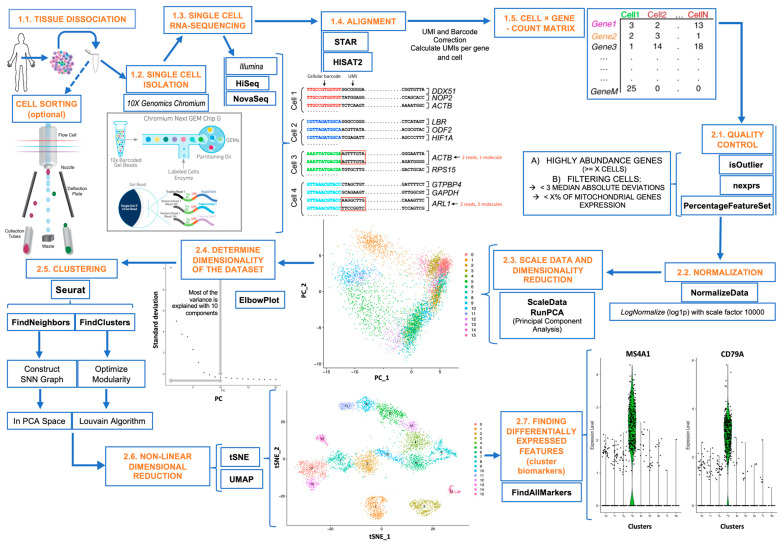
Workflow applied to the analysis of the single-cell RNA-seq datasets studied in this work. The first part (from 1.1 to 1.5) corresponds to the experimental steps from the biological samples till obtaining the raw expression counts per cell and per gene. These steps include the following: the single-cell isolation (using 10x Genomics Chromium platform), the single-cell RNA sequencing (using Illumina sequencers HiSeq or NovaSeq), the quantification of the reads, the alignment of the reads to the reference genome or transcriptome, and the calculation of the raw counts data matrix per cell and per gene. The second part (from 2.1 to 2.7) includes the actual analytical bioinformatics section of the workflow, starting with quality control and filtering, normalization, scaling, and dimensionality reduction, clustering for the identification of different cell populations, and generation of the Seurat single-cell object containing all the output data (i.e., the quantification of gene expression per cell, the clusters or groups of cells found, and the phenotypic information of the samples). We applied the second part of the workflow described to generate the Seurat objects for each dataset.

We first prepared a bioinformatics workflow for scRNA-seq data analysis, shown in Figure 1, and applied it to the three collected datasets. The analysis was run using the expression data of different selected gene lists: (i) the CD list of 369 human genes known to encode for CD markers, taken from the Human Protein Atlas (List 1, with 369 genes); (ii) a list of membrane-associated genes that included all the 369 CD markers plus an additional set of 63 genes that encode for membrane proteins (List 2, with 432 genes); (iii) the list of all the genes that were detected as expressed in each of the three scRNA-seq datasets analyzed (List 3, containing a different number of genes in each dataset).

**Figure 2:**
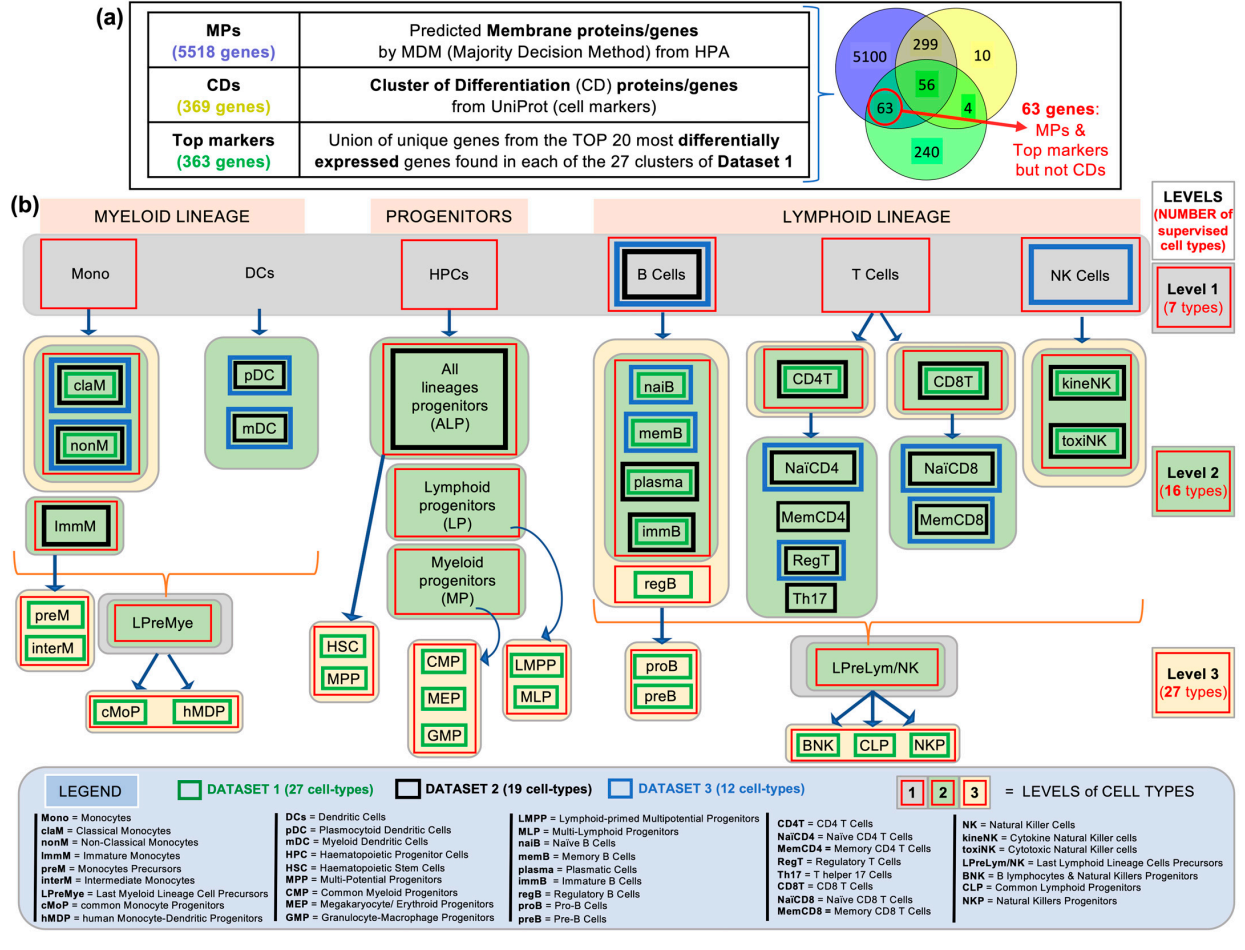
Scheme showing (**a**) the lists of human genes that are used in this work, indicating their size and the overlap between them: 5518 genes predicted to encode membrane proteins; 369 genes encoding known CD markers; and 363 genes corresponding to the union of unique genes from the TOP 20 most differentially expressed genes found in each of the 27 clusters of Dataset 1. The Venn diagram shows the intersections between these 3 sets of genes. Part (**b**) of this figure schematically shows all the hematological and immune cell types that we worked with in this study and that were detected in the 3 datasets (Datasets 1, 2, and 3). The cell types identified in each of these datasets are marked with a colored square: *green* squares for Dataset 1, which includes 27 cell types; *black* squares for Dataset 2, which includes 19 cell types; and *blue* squares for Dataset 3, which includes 12 cell types. The cells are organized into lineages: myeloid cells, progenitor cells, and lymphoid cells; and from less specific to more specific cell types (from top to bottom). The legend at the bottom gives the names of all the specific cell types studied. Finally, colored background panels with a red square inside are included in the figure to mark the cell types studied at different levels of dissection or cell type separation. Thus, three main levels are considered: Level 1, which includes 7 different cell types in the *grey*-colored panels; Level 2, which includes 16 cell types in the *green*-colored panels; and Level 3, which includes 27 different cell types and subtypes in the cream colored panels. This largest number of cell types (27 in Level 3) is the same defined in Dataset 1, because that study included experimental validation of the different cell populations and was therefore used as a reference in this work.

The set of 63 genes included in List 2 was obtained from the intersection of two lists: (i) the list of 363 genes generated as the union of unique genes from the TOP 20 most differentially expressed genes found in each of the 27 clusters of Dataset 1 (obtained with FindAllMarkers, Seurat function); with (ii) the list of 5518 genes predicted to encode membrane proteins (MPs) by MDM (majority decision method) but that were not included in the list of 369 CD markers. The intersection of these two gene lists yielded a new list of 63 genes (as shown in Figure 2a, intersection of 5518 MP genes with 363 top marker genes: MPs and top markers but not CDs).

Using three independent scRNA-seq datasets and following the workflow described in Figure 1, we performed a series of analyses to determine how many different cell types and subtypes could be identified in each dataset using the expression of the three gene lists described at the beginning of this section (i.e., the MPs, the CDs, and the top markers reported in Figure 2a). In Figure 2b, we present a graphical scheme including all the different hematological and immune cell types identified in the single-cell data, organized into three lineages: myeloid, lymphoid, and progenitors, starting with six general cell groups: monocytes (Mon), dendritic cells (DCs), B lymphocytes (B cells), T lymphocytes (T cells), and natural killer cells (NK cells). Each of these major cell groups contains several different cell types or subtypes, which are shown in a second layer in Figure 2b. The names of all these cell types are given in the LEGEND in Figure 2. All different cell populations identified in the analyses of each one of the three single cell datasets studied (Datasets 1, 2, and 3, reported in Table 1) are marked with a colored square in Figure 2b, and they are the following: 27 cell types found in Dataset 1; 19 in Dataset 2; and 12 in Dataset 3. The largest number of cell types included in this analysis, 27, was found in Dataset 1. Furthermore, these cell types were experimentally validated in the work published by Xie et al. (2020) [9]. Therefore, we used Dataset 1 as a reference for the identification of cell types at the deepest granularity (i.e., the deepest level of cell class separation and analysis). We also used two other levels with a smaller number of cell types: Level 2, which distinguishes 16 cell types; and Level 1, which distinguishes only 7 more general cell types.

### 2.3. Comparative Analysis of scRNA-Seq Datasets of Blood and Bone Marrow Mononuclear Cells

**Figure 3:**
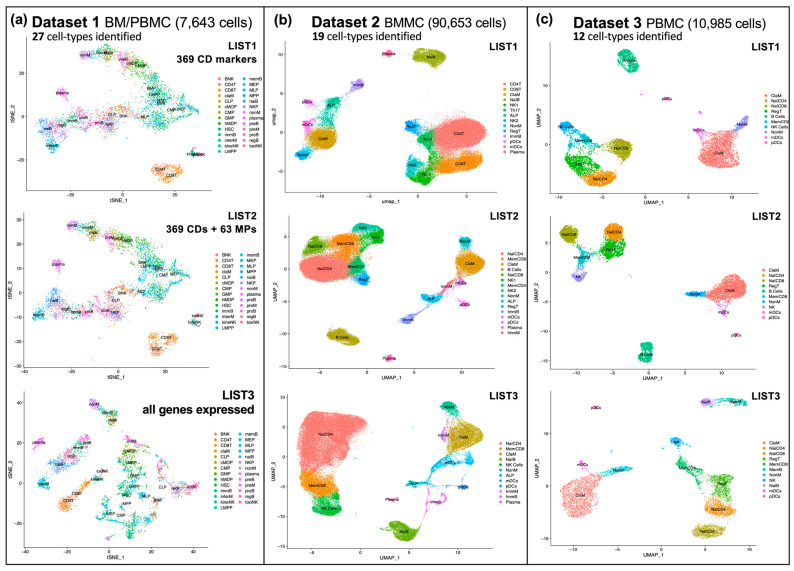
Single-cell tSNE and UMAP maps of three complementary sample cell sets obtained from peripheral blood mononuclear cells (PBMCs) and bone marrow mononuclear cells (BMMCs). (**a**) Three tSNE plots corresponding to the analysis of Dataset 1, which contains 7643 cells and identifies clusters corresponding to 27 different cell types. The three plots include clusters of different cell populations (i.e., different cell types or subtypes) generated using different numbers of genes: 369 CD markers (gene List 1); 369 CD markers plus 63 genes encoding membrane proteins (List 2); and all expressed genes detected in the cells of this dataset (List 3). (**b**) Three UMAP plots corresponding to the analysis of Dataset 2, which contains 90,653 cells and identifies clusters corresponding to 19 different cell types. The three plots were generated in the same way as indicated above, using different numbers of genes: List 1; List 2; and List 3. (**c**) Three UMAP plots corresponding to the analysis of Dataset 3, which contains 10,985 cells and identifies clusters corresponding to 12 different cell types. The three plots were generated in the same way as indicated above, using different numbers of genes: List 1; List 2; and List 3.

A comparative analysis of three independent scRNA-seq datasets of peripheral blood mononuclear cells (PBMCs) and bone-marrow mononuclear cells (BMMCs) isolated from healthy donors was performed using the bioinformatic workflow presented in Figure 1. The visualization of the single-cell maps obtained for each of these datasets is shown in Figure 3, which also indicates the number of different cell types identified in each set.

The different cell clusters identified in the analyses of the three datasets shown in Figure 3 were found using the expression signal detected in the cells corresponding to different lists of genes. As the cells under investigation were hematological and immune cells, the first search was carried out using the list of known CD markers. Cluster of differentiation (CD) markers are cell surface molecules or antigens used in immunology and cell biology to identify and characterize different cell types. This list is mainly composed of proteins, so we took it from the Human Protein Atlas database (https://www.proteinatlas.org/search/protein_class:CD+markers, accessed on 16 December 2021), which provided us with a list of 369 proteins encoded by genes (List 1 in Figure 3, included in Supplementary Table S1). The second gene list we used had 432 genes (List 2), which was composed of 369 CD markers plus 63 genes encoding membrane proteins, as mentioned above. The third gene list used (List 3) contains all genes that were found to be expressed (i.e., detected) in the three BM/PBMC datasets, which were the following: 19,813 genes in Dataset 1; 33,660 genes in Dataset 2; and 36,601 genes in Dataset 3. As can be seen from the tSNE and UMAP plots in Figure 3, the granularity of the single-cell maps is marked by the number of different cell populations found in each dataset and is equal to the number of clusters identified in each set. The number of clusters found was the following: 27 in Dataset 1, 19 in Dataset 2, and 12 in Dataset 3; and these numbers of clusters correspond to the same number of cell types described in Figure 2. At the same time, the granularity within each dataset increases as the number of genes included in the analyzed expression data increases.

### 2.4. Evaluation of the Clustering with Silhouette Using Different Gene Lists

We evaluate the clustering power of the three gene lists in the three independent datasets, taking into account the highest ability to separate and distinguish different groups of cells when applying the Silhouette algorithm. The results of this analysis are presented in Supplementary Figure S1, which shows in nine plots the output of the clustering algorithm Silhouette for each one of the three datasets and each one of the three gene lists. The number of distinct groups found in each case were the following: (i) Dataset 1, 19 clusters with List 1, 18 clusters with List 2, and 28 clusters with List 3; (ii) Dataset 2, 15 clusters with List 1, 17 clusters with List 2, and 20 clusters with List 3; (iii) Dataset 3, 11 clusters with List 1 and also with List 2 and 12 clusters with List 3. The value of the mean Silhouette width (MSW), as a measure of the separation and definition of the clusters, is given for each clustering in Supplementary Figure S1, and it was greater than 0.5 in four cases: in Dataset 1 with List 3 (0.51) and in Dataset 3 with List 1 (0.53), List 3 (0.57), and List 3 (0.54). It is clear that the ability to separate the cells into different groups depends on the number of genes used in the clustering, as these are the features that characterize the cell types, but also on the quality of the transcriptomic signal obtained in each dataset. It is also evident that the smaller the number of clusters identified, the easier it is to optimize the separation between clusters.

In Dataset 1, the average Silhouette widths (MSW = 0.49, 0.48, 0.51) did not show high differences between the use of the three gene lists tested. On the other hand, Dataset 2 and Dataset 3 showed their highest values using List 2, which had 432 genes (MSW = 0.39 and 0.57, respectively). These results showed that the ability to discriminate cell types by adding membrane protein genes (which were included in List 2 with respect to List 1) increased the separation capacity compared to using only the CD genes (List 1). Furthermore, the results of this analysis with the Silhouette algorithm also show that the number of cell types found (i.e., the number of distinct cell populations or distinct groups of cells) is not dependent on the number of cells tested or the number of genes detected in the single-cell technology, because Dataset 1 has fewer cells (7642 cells) and fewer genes (19,813 genes) than Dataset 2 (with 90,653 cells and 33,660 genes), but Dataset 1 has many more clusters and a better separation of the clusters than Dataset 2. This also indicates that the quality of the obtained single-cell transcriptomic profiles is more important than the number of cells and genes.

### 2.5. Finding Gene Markers for Specific Cell Types and Subtypes Identified in the Single-Cell Datasets

**Figure 4:**
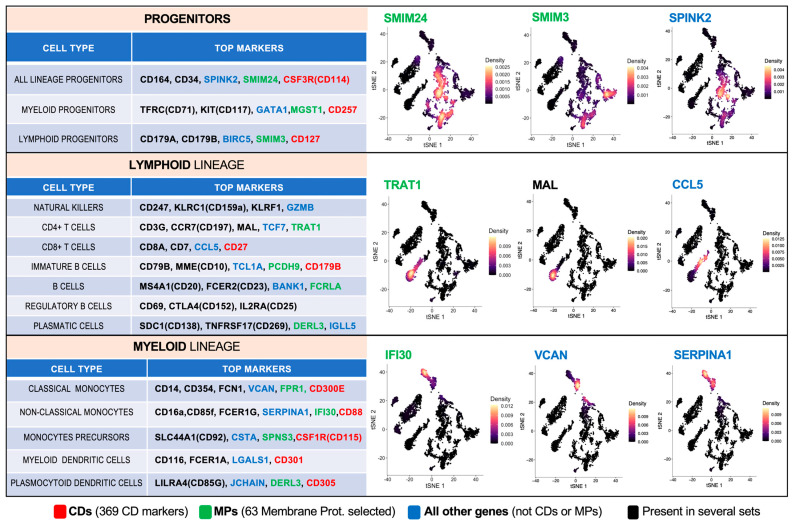
Tables on the left: selected top markers for 15 cell types: (i) 3 types of progenitor cells; (ii) 7 types of lymphoid lineage cells; and (iii) 5 types of myeloid lineage cells. Right panels: for each group of cell types, three tSNE plots corresponding to Dataset 1, showing the expression of 3 selected genes (marked in red or yellow according to the scales included in each plot). The figure also shows, in red, green, or blue, the source list of the genes (i.e., the set in which the genes are included: the CD set, the MP set, or the set that is all other genes but not CDs or MPs).

After identifying the main clusters or groups of cells found in the analyses of the three datasets studied, we ran a series of functions using the Seurat library to find the main gene markers of each cell cluster. These markers are chosen because they show, for each cell cluster, a significant differential expression with respect to all the other cells of the single-cell map. In this way, we found the genes included in Figure 4, which shows a set of top markers for fifteen cell types: three progenitor cells; seven lymphoid lineage cells; and five myeloid lineage cells.

Following a similar strategy described above in Figure 4 for the identification of gene markers associated with each cell type, we performed a more exhaustive search for markers using the highest number of different cell types and subtypes identified in our study, which was 29: (i) 27 cell types derived from Dataset 1 (as shown for Level 3, the deepest level, described in Figure 2); plus (ii) 2 cell types corresponding to dendritic cells not detected in Dataset 1 but detected in Datasets 2 and 3 (pDC and mDC in Figure 2). The full name and lineage of these 29 cell types and subtypes are presented in Supplementary Table S2, where it can be seen that 2 correspond to stem or multipotent progenitors (HSC and MPP), 5 to lymphoid progenitors (BNK, CLP, LMPP, MLP, and NKP), another 5 to myeloid progenitors (cMoP, CMP, GMP, hMDP, and MEP), 12 correspond to cell types of the lymphoid lineage, either B cells, NK cells, or T cells (immB, memB, naiB, preB, proB, regB, kineNK/NK1, toxiNK/NK2, plasma, CD4T, CD8T, and pDC), and finally 5 correspond to cell types of the myeloid lineage (claM, interM, nonM, preM, and mDC).

Using these 29 cell types, we interrogate the clusters obtained in the analysis of the single-cell data (27 types found in Dataset 1 and 2 types from Dataset 3) to obtain the top 30 gene markers that are most differentially expressed (i.e., most active) in each of these cell types compared to the rest of the cell types in the data. This was performed using the *FindAllMarkers* function of the Seurat algorithm. The results of this search are provided in Supplementary Table S3, which includes, for each of the 29 cell types, the 30 genes that best mark that cell type, ranked by adjusted *p*-values (obtained in the statistical contrast of the cells assigned to each cell type versus all other cells). The analysis also includes, for each cell type, the percentage of cells expressing each gene marker in the cluster assigned to that type versus the average percentage of expression of that gene in all other clusters. In total, this search for the best markers of these 29 cell types yields a set of 596 unique genes that will be further compared with other gene sets used to identify hematological and immune cell types.

### 2.6. Machine Learning Comparative Evaluation of the Cell Type Gene Markers Found

After evaluating the ability to separate and distinguish our cells in each dataset, we tested how our gene signature (i.e., the set of 596 identified gene markers) was able to distinguish different cell types and subtypes compared to other gene signatures. We carried this out using the Random Forest algorithm within Dataset 1 as it was supervised, and we compared the performance of four gene signatures: (1). the set consisting of the top 30 CDs that best identify the clusters in Dataset 1 (218 unique genes); (2). the set consisting of the top 30 CDs together with membrane protein genes (MPs) that best identify the clusters in Dataset 1 (281 unique genes); (3). the set of 596 unique gene markers we identified (described above); (4). the set of 547 genes included in the LM22 signature of CIBERSORT, which is a standard reference set for the identification of hematological and immune cells [12,13]. We tested these four signatures at three different “levels” of cell type and subtype separation granularity: Level 1, which includes 7 cell types; Level 2, which includes 16 cell types; and Level 3, which includes 27 cell types (the cell types included in each level are described in Figure 2). 

**Figure 5:**
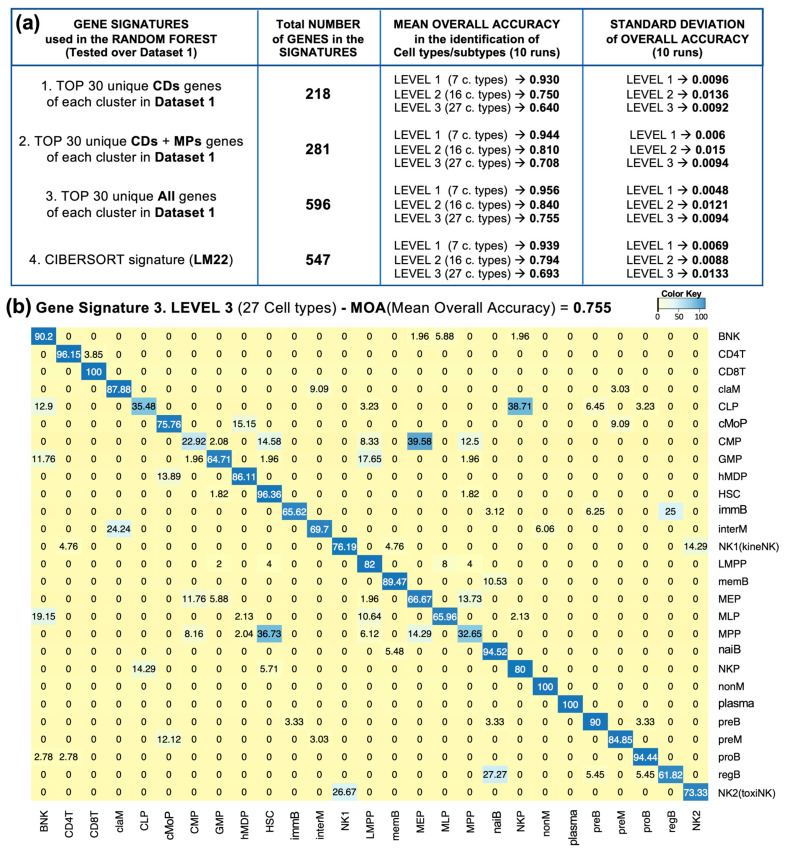
Summary of the Random Forest results obtained with the 4 gene signatures tested: (**a**) Table describing the 4 gene sets used in the Random Forest and the results, at 3 levels, obtained for overall accuracy (mean and sd); (**b**) Confusion matrix obtained after 10 runs of the test to predict the cell-type label of each of the 27 clusters of cells present in the single-cell analysis of Dataset 1.

In all cell type levels of prediction, the highest accuracy (i.e., the best mean overall accuracy, MOA = 0.96 for 7 cell types of Level 1 and 0.84 for 16 cell types of Level 2) was obtained with the signature produced by selecting the top 30 of all genes (i.e., the signature of 596 genes) (Figure 5a). And the second best was obtained with the signature produced by selecting the top 30 CDs and membrane protein genes of each cluster (MOA = 0.94 for Level 1 and 0.81 for Level 2), which is better than the widely used LM22 signature. These results once again showed that deep analysis of single-cell data can provide many new cell-specific gene markers and that the use of some extra genes (such as those predicted to be membrane proteins by MDM), added to the list of CDs, provided a set of markers that were quite accurate for identifying hematological and immune cell types (Figure 5).

Figure 5a shows the results of the comparative evaluation, performed using Random Forest, of the predictive power of each one of the four gene signatures (four sets of gene markers) obtained in the identification of the cell types and subtypes present in Dataset 1. The comparisons are performed considering 7 different cell types, 16 cell types, and 27 cell types, corresponding to three levels of granularity. The accuracy (MOA) corresponding to each set and each level is provided. Since the Random Forest test was run 10 times, Figure 5b shows a heatmap of the binary matrix including, for each of the 27 cell types, the times that the cells of a given cluster are correctly assigned to the known label (i.e., the times that the cells of a cluster are assigned to the correct cell type).

The figure shows that most cell types, such as CD4T, CD8T, classical and non-classical monocytes (claM, nonM), plasma cells, etc., are well identified, with a correct classification rate above 85. In contrast, the cell types that are most confused are those that are closer in lineage, such as subtypes of B cells (for example, regulatory B cells, regB, and naive B cells (naiB)) or subtypes of progenitors (such as megakaryocyte/erythroid progenitors, MEPs, confused with multipotential progenitors, MPPs; or megakaryocyte/erythroid progenitors, MEPs, with common myeloid progenitors, CMPs).

### 2.7. Analysis of Cell Trajectories in the Single-Cell Map to Reveal Cellular Relationships and Identify Different Lineages

Another complementary analysis that we can perform on the single-cell data of the hematological and immune cell sets studied here is the analysis of the trajectories that may be present in the cell maps. This analysis, performed using the *TSCAN* algorithm [14], explores the data to find progressive changes in the expression profiles of specific genes along the groups or clusters of cells identified in the maps.

**Figure 6:**
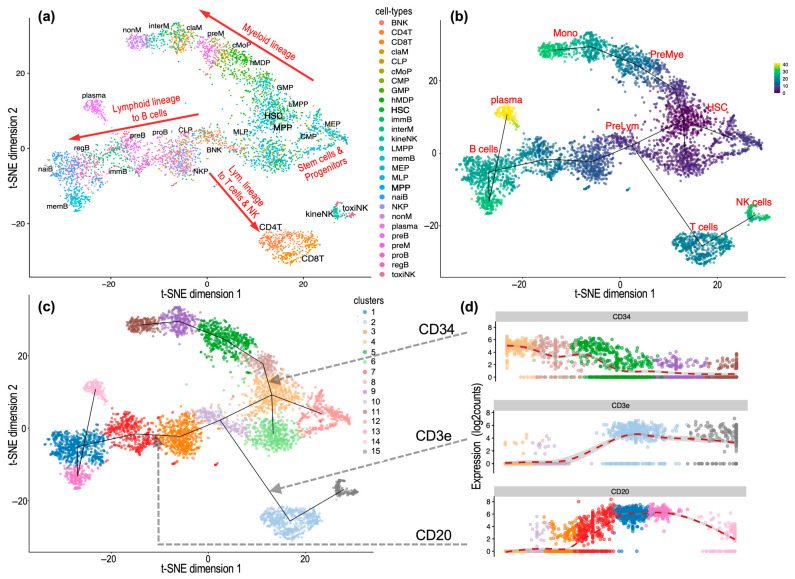
Trajectory analysis of the cells from supervised Dataset 1. (**a**) Single-cell t-SNE map showing populations of 27 cell types and subtypes. (**b**) The same single-cell t-SNE map with a color gradient indicating the differentiation trend: from dark purple for progenitor phenotypes to light green and yellow for differentiated phenotypes. (**c**) The same single-cell t-SNE map, now with the major cell clusters (15 groups) highlighted in color and the trajectories found by *TSCAN* indicated by black lines. (**d**) Expression profiles of three gene markers (CD34, CD3e, and CD20) that reflect the trajectories found corresponding to the myeloid lineage, the lymphoid lineage till B and plasma cells, and the lymphoid lineage till T cells and NK cells.

Figure 6 shows this analysis of trajectories upon the single-cell data of Dataset 1. This dataset was supervised, and the original study [9] provided the experimental identification of multiple cell types and subtypes of hematological and immune cells, followed by the corresponding assignment of these types to 27 cell populations and cell clusters in the single-cell map. This mapping is shown in Figure 6a, and thanks to the fact that the dataset includes several progenitors of the main lineages (myeloid and lymphoid lineage), as well as most of the differentiated cells of these lineages (such as six subtypes of B cells or four subtypes of monocytes), we obtain three clear trajectories derived from the expression profiles of the cells, which reflect very well the evolution of the cells from populations of progenitors to populations of differentiated cells.

The three main trajectories found in the analysis of the single-cell map of Dataset 1, shown in the t-SNE plots (Figure 6a–c), reflect that the expression profiles of the cell populations move or evolve from stem cells (HSCs) to monocytes (Mon), revealing the myeloid lineage; move from stem cells (HSCs) to B lymphocytes (B cells and plasma cells), revealing the B lymphoid lineage; and move from stem cells (HSCs) to T lymphocytes (T cells and NK cells), revealing the T-lymphoid lineage.

## 3. Discussion

We conducted a comprehensive integration and analysis of three single-cell RNA sequencing datasets derived from human peripheral blood and bone marrow cells. Our investigation focused on identifying and evaluating three gene sets (List 1: 369 CD marker genes; List 2: 432 genes, CD markers plus 63 membrane proteins; and List 3: all expressed genes detected in each dataset) to establish robust and functional signatures for distinct cell types and subtypes. The high resolution of the scRNA-seq technique enabled us to uncover novel insights into the expression profiles of blood and immune cells that could refine and expand the gene markers currently employed for isolating and studying these cellular populations.

Our primary objective was to assess the ability of the different gene sets to distinguish cell types. Analysis using Silhouette scores revealed that List 2, comprising 432 genes, demonstrated superior discrimination of hematological cell types. This suggests that the additional 63 genes in List 2, predicted to encode membrane proteins by MDM, significantly enhance cell-type resolution. Notable markers include established genes such as DERL3 for plasma cells, KLRF1 for NK cells, and FCRLA for B cells [15,16,17]. Additionally, we identified potentially novel markers with high specificity and limited prior characterization, such as MGST1, SMIM3, and SMIM24 for progenitor cells or FPR1, IFI30, and SPNS3 for myeloid cells. These findings offer promising avenues for further investigation into their roles in cell identity and function.

Regarding progenitor cells, SMIM24 has been previously associated with a stemness blood phenotype [16], while the roles of SMIM3 and MGST1 in this context remain less clear. These two markers were observed to indicate specific lineage immaturity, corresponding to lymphoid and myeloid lineages, respectively. These progenitor cell populations are not well characterized yet [17], emphasizing the importance of the information provided by these novel markers. We saw this fact in Dataset 1, as the algorithms were unable to achieve reliable isolation, and the clustering did not correspond to the cell types assigned by FACS. This indicates that, in addition to the lack of well-defined markers, the precise identities of the cell types, such as CMPs (common myeloid progenitors) or GMPs (granulocyte–macrophage progenitors), remain unclear. Evidence suggests that distinct human myeloid progenitor populations give rise to neutrophil/monocyte and mast cell/basophil/eosinophil lineages, with the latter populations clustering together and segregating from neutrophil/monocyte lineages [18]. In our analysis, this segregation was reflected by the clustering of CMPs and MEPs (megakaryocyte–erythroid progenitors) together, while GMPs and cMOPs (common monocyte progenitors) formed a separate cluster, representing precursors to monocytes and neutrophils. Moreover, the existing FACS markers for these cells showed a poor correlation with their transcriptomic profiles. These findings highlight the need for further studies to identify robust gene signatures that can better distinguish the subtypes of hematopoietic progenitor cells.

Moving to myeloid cells, the novel membrane markers proposed (FPR1, IFI30, and SPNS3) did not show a clear relationship with the cell types we assigned when compared to information from the Human Protein Atlas [19]. Nevertheless, the marker assignments derived for these cells enabled successful isolation, as discussed in the Section 2 regarding the establishment of these robust signatures. This finding was further validated using the Random Forest prediction method, along with Silhouette score analysis, which yielded the highest values for these myeloid cells. These results confirm that myeloid cells are the best-separated populations with the most distinctive gene signatures.

In our clustering analysis, one of the most notable findings involved the CD4 marker in lymphoid cells. Across all datasets, we observed that CD4 was more highly expressed in monocytes than in the commonly designated CD4+ T lymphocytes. Interestingly, in both datasets, only about 10% of these T lymphocytes expressed this marker. This finding is significant, because despite the low expression of CD4 in these cells at the transcriptomic level, the presence of the CD4 protein on the cell membrane remains high. Currently, protocols for isolating lymphoid cells for clinical applications, such as CAR-T cell development, tend to focus on more functional markers, such as those identified and used in our study (e.g., CD27 and CD8) [20,21]. This highlights the importance of using robust and functionally relevant markers for both research and therapeutic purposes.

A similar observation was made with NK cells, where we consistently isolated the same activator and repressor markers across datasets, enabling the algorithms to identify two distinct clusters or populations. The conventional FACS-based classification of NK cells relies on the CD56 and CD16 markers, which we confirmed in our datasets: one cluster exhibited high CD56 expression with lower levels of CD16a, while the other showed higher CD16a expression with no CD56 expression [22]. The significance of our findings lies in the additional markers associated with these two profiles. For the high-CD56 population, markers such as KLRC1 and KLRF1 were prominent, whereas the non-CD56 population was associated with markers like KIR2DL3 and KIR3DL2. These markers define two functional NK cell populations: the low-CD56 population, characterized by high cytotoxic activity, and the high-CD56 population, which exhibited a more immunomodulatory profile with reduced cytotoxicity [22]. This distinction has important implications for isolating NK cells for the development of CAR-NK therapies. In particular, selection of NK cells based on functional markers such as KIR2DL3 or low-CD56 expression for cytotoxic activity may be advantageous, as this behavior was consistently observed in all cases.

To isolate these markers, we identified the most differentially expressed markers for each cluster and utilized trajectory analysis in Dataset 1 to track how their expression profiles changed along the differentiation trajectory (adding the pseudotime hyperparameter). This approach provided valuable insights for precisely delineating each subpopulation and constructing robust gene signatures. To evaluate the performance of CD markers, we compared the cell-type assignment capacity of our markers against other gene lists (Lists 2 and 3), which also included CD markers. Using Random Forest algorithms, we observed that the signature incorporating the 63 additional membrane genes outperformed others. This confirmed the effectiveness and selection strength of this human surface marker list (List 2) in our single-cell transcriptomic datasets. Importantly, this list could serve as the foundation for defining a new human cluster of differentiation (CD) for peripheral blood and bone marrow cell populations. This finding is particularly noteworthy, as previous studies of CD markers and membrane proteins have primarily relied on methodologies such as flow cytometry, mass spectrometry, or RNA-seq [23,24,25]. By contrast, our single-cell approach provides a novel perspective and enhanced resolution for identifying and characterizing these markers.

Additionally, we investigated several key non-membrane-associated markers from List 3. One such marker, TCF7, is particularly notable, having been previously characterized for its expression in naïve and immature T cells. TCF7 plays a crucial role in the development of memory phenotypes within these T-cell populations [26]. In this way, TCF7 is a well-known marker for CD8 memory T cells, but it can also indicate immature T cells. This reflects an inherent complexity when using certain genes as exclusive markers, as their expression may be present in more than one cell population, making it difficult to accurately and specifically interpret each of the clusters assigned to immune cells or difficult to identify a specific population, since multiple cell types share functional pathways or molecular features. In our study, fully aware of these complexities, we did not use a single marker for a cell type but rather applied an analytical approach that identifies multiple markers for each cluster combined with multiple clustering algorithms. We also identified surface markers indicative of stemness, including SPINK2, which was specifically expressed in hematopoietic stem cells (HSCs), the most undifferentiated cell populations, in a manner analogous to CD34 expression [27]. Furthermore, GATA1 was identified as a marker of myeloid–erythroid progenitors [28].

Despite the valuable insights provided by our study, there are several limitations to consider. First, the cells analyzed were isolated from bone marrow and peripheral blood, so the gene signatures identified may not be representative of all immune cells present in other tissues, such as liver or intestine. In addition, certain cell populations were only present in a single dataset, highlighting the need for further validation. Furthermore, the absence of specific cell types in certain datasets led to the assignment of non-specific markers to related lineage populations that were present. Finally, future work will be required to validate the novel markers identified in this work for specific cell types and cell populations. In fact, as a complement to the scRNA-seq data presented here, we understand that practical confirmation of the gene signatures found will be required using cellular techniques based on protein recognition, such as flow cytometry (FC) or fluorescence-activated cell sorting (FACS). These techniques use specific labeling with antibodies and are the standard primary approaches to validate markers that may become novel CDs. In addition, we plan to provide complementary validation to the results presented here using new scRNA-seq datasets that will allow refinement and extension of our findings.

Indeed, incorporating datasets from individuals in diverse physiological and pathological conditions—such as fasting, diabetic, aging, or cancer-affected subjects—would significantly strengthen the applicability and robustness of the proposed markers across a broader range of contexts. This approach would allow us to evaluate whether the identified markers reliably distinguish cell populations under varying biological states and pathologies. The inclusion of such datasets would provide a clearer understanding of the stability and relevance of the markers under different physiological conditions, potentially leading to more comprehensive marker panels with translational value for clinical applications. Furthermore, this would help refine the identified clusters by accounting for variability introduced by disease states, which is a key factor in achieving robust cell-type classification. Importantly, recent studies have demonstrated that scRNA-seq profiling of CD markers enhances immune cell classification and improves patient stratification for immunotherapy. Sun et al. [29] showed that single-cell transcriptomics can identify novel immune checkpoints and biomarkers that predict responses to immune checkpoint inhibitors. This personalized approach allows for distinguishing between responders and non-responders, improving treatment outcomes. Moreover, scRNA-seq has uncovered immune escape mechanisms that guide the development of next-generation immune therapies, reinforcing the relevance of CD marker profiling in clinical oncology. Additionally, specific CD marker polymorphisms have been associated with prognostic outcomes in hematological malignancies, emphasizing their utility in personalized treatments [4]. These findings highlight the importance of validating our proposed gene signatures in clinical settings. Expanding marker validation efforts through proteomic approaches or disease-state datasets could strengthen diagnostic precision and improve therapeutic interventions in diseases such as cancer, autoimmune disorders, and infectious diseases.

## 4. Materials and Methods

### 4.1. Single-Cell Data Collection

Three independent single-cell RNA sequencing datasets were collected containing single-cell gene expression data from hematopoietic mononuclear cells (MNCs) from bone marrow (BM) and peripheral blood (PB) obtained from healthy adult human donors. The datasets are presented in Table 1, indicating the source [9,10,11], the public repository from which they come, the platform used for the mRNA expression analyses, the number of cells separated and tested in each case, and the number of samples used in each dataset. In all cases, the cells were isolated using the 10x Genomics Chromium platform for single-cell separation, and the transcriptomic sequencing (scRNA-seq) was performed using the following platforms: Illumina HiSeq 3000, HiSeq 4000, and NovaSeq 6000.

The first dataset (Dataset 1) contains hematopoietic mononuclear cells from the bone marrow (BM) and peripheral blood (PB) of 21 healthy adult donors. The data from this set were supervised by analyzing the samples with FACS (fluorescence-activated cell sorting) to provide 32 well-defined cell types. The expression count matrix from this dataset included 7643 cells and 19,813 gene transcripts, and was filtered to remove 122 low-quality cells, using a cut-off threshold for the cells that presents less than 1000 detected protein-coding genes. The dataset was obtained from the NCBI Gene Expression Omnibus (GEO) database (https://www.ncbi.nlm.nih.gov/geo/GSE149938, accessed on 12 December 2024) corresponding to accession number GSE149938. The second dataset (Dataset 2) was generated using bone marrow mononuclear cells (BMMCs) from 20 healthy donors (10 males and 10 females). This dataset was unsupervised, and the final count matrix has 76,684 cells and 33,660 transcripts. The filtering thresholds used with this dataset were at least 500 transcripts per cell and less than 8% mitochondrial RNA content. This dataset corresponds to accession GSE120221 in GEO (https://www.ncbi.nlm.nih.gov/geo/GSE120221, accessed on 12 December 2024). The third dataset (Dataset 3) was generated with peripheral blood mononuclear cells (PBMCs) from a healthy donor. This one was also unsupervised, and the final count matrix has 9238 cells and 36,601 transcripts. The filtering thresholds used in this case were more than three median absolute deviations (MADs) to filter outlier cells and transcripts and filtering of cells with more than 10% mitochondrial RNA content. This dataset was obtained from the 10x Genomics data resource (https://www.10xgenomics.com/resources/datasets/, accessed on 12 January 2022) and corresponds to the set described in reference [11]. Finally, before starting the analysis of these datasets the erythrocytes, if present, were filtered out.

### 4.2. Bioinformatic Analyses

#### 4.2.1. Cell and Gene Filtering

The whole bioinformatic analysis developed in this work was produced with R Software v4.0.3 (R Core Team 2017). R is a programming language and environment for statistical computing used for the whole analyses done in this work (R Foundation for Statistical Computing, Vienna, Austria. URL https://www.R-project.org/). Once we obtained the count matrix, the first step was the quality control to filter out and delete genes and cells based on different criteria: less expressed genes, expression value outliers based on MADs, and the percentage of mitochondrial RNA content. For these steps, we employed the functions nexprs and isOutlier of scater package [30] and PercentageFeatureSet from Seurat package [31] (2.1-Figure 1).

#### 4.2.2. Normalization

From this point, all the functions mentioned below are from Seurat package (version 4.0) [31]. For normalization, we employed the function NormalizeData in all datasets (2.2-Figure 1), and we specifically performed log normalization using 10,000 as the scale factor.

#### 4.2.3. Scaling and Linear Dimensionality Reduction

We performed the scaling and the linear dimensionality reduction of the data employing principal component analysis (PCA). The functions that we used were ScaleData (scale.max = 10) and RunPCA (2.3-Figure 1). To determine the dimensionality of all datasets, we represented a dot chart that indicated the percentage of the variability explained by each principal component (2.4-Figure 1).

#### 4.2.4. Clustering

The clustering was performed with the functions FindNeighbors and FindClusters, using the shared nearest neighbor (SNN) algorithm [32] with PCA as dimensionality reduction and the original Louvain method [33] as modularity optimization tool, respectively (2.5-Figure 1). The following parameters were used for these clustering analyses: 10 random starts, 10 maximum iterations per start, and 1.2, 0.6, and 0.6 as the specific resolutions applied over all gene signatures for each of the three datasets, respectively. The clustering visualization and the checking of the grouping of cells were performed using non-linear dimensionality reductions. Specifically, we employed two different methods: UMAP [34] and t-SNE [35] (2.6-Figure 1).

#### 4.2.5. Marker Genes Selection

The identification of the most differentially expressed genes (DEGs) in each cell population or cluster was determined by FindAllMarkers function employing the Wilcoxon rank sum test. The parameters used were min.pct = 0.25; logfc.threshold = 0.25; only.pos = TRUE (2.7-Figure 1).

#### 4.2.6. Trajectory Analysis

For the trajectory analysis, we used the algorithms of TSCAN package [14]: createClusterMST, reportEdges, mapCellsToEdges, orderCells, and testPseudotime functions. We represented and calculated the minimum spanning tree (MSS) on cluster centroids, we calculated the distances and edges between the different clusters, and finally, we obtained significant differences concerning the pseudotime hyperparameter, illustrating the cellular progression across time.

#### 4.2.7. Evaluation of Cell-Type Assignment

Random Forest [36] is a machine learning technique or classification algorithm based on the construction of many decision trees. We used it for the evaluation of the classification power of the different genomic signatures (employing the following as parameters: importance = TRUE and nt = 10,000). We selected a random 30% of cells of each cell population for the training and a random 30% of each cell type (not including the 30% of the training set) for the validation. Furthermore, we carried out cross-validation by performing the same analysis 10 times.

#### 4.2.8. Evaluation of Cell Clustering

Silhouette [37] is an algorithm employed for the interpretation and validation of a cluster analysis. We employed it to evaluate which gene signature was able to discern or distinguish cell types in a better way in the non-linear dimensionality reduction space. Specifically, the function that we employed for this clustering evaluation was silhouette from cluster package [38], and its output value depends on the tightness and separation of the found populations.

#### 4.2.9. Cell-Type Annotation

The annotation of cell types was performed semi-automatically using multiple approaches to ensure accuracy and biological relevance. Specifically, we used tools such as the HumanPrimaryCellAtlas from celldex library [39], as well as databases like the Human Protein Atlas [19] to validate key markers. Additionally, we consulted the original research articles and dataset analyses where cell-type annotations were previously conducted, ensuring consistency between our work and previously established annotations.

## 5. Conclusions

As a general conclusion of this work, we would like to emphasize the scope of the study we conducted, which aimed to elucidate and differentiate various hematological and immune cell types using single-cell markers and to establish robust gene signatures for the cell populations studied. This approach has significant potential to enable more precise and functional cell-specific isolation in various research studies and clinical trials involving hematological and immune cells. Indeed, the cell-type-specific signatures that we have found and included in this article provide a valuable resource for further research in this area. In addition, our results may prove valuable in the diagnosis of hematological malignancies [4] as well as solid tumors, particularly when investigating the complex cellular composition of the tumor microenvironment and the gene signatures of infiltrating leukocytes [40] present in the disease niche.

## Figures and Tables

**Table 1:** Information of the datasets used for the single-cell analysis. BM = bone marrow; PBMCs = peripheral blood mononuclear cells; BMMC = bone marrow mononuclear cells.

Datasets	Authors *Journal* (Year)	Year and [Ref.]	Public Repository	Platform Used	Number of Cells	Number of Healthy Donors	Source Tissue	Supervised Data (FACS)
**Dataset 1**	Xie et al. *Natl Sci Rev* (2021)	2020 [9]	GEO: GSE149938	Illumina HiSeq 4000 (Homo sapiens)	7643	21	BMMC/ PBMC	Yes
**Dataset 2**	Oetjen et al. *JCI Insight* (2018)	2018 [10]	GEO: GSE120221	Illumina HiSeq 3000 (Homo sapiens)	90,653	20	BMMC	No
**Dataset 3**	10x Genomics (2020)	2020 [11]	10k PBMCs from a healthy donor	Illumina NovaSeq 6000 (Homo sapiens)	10,985	1	PBMC	No

## Data Availability

The raw data of the single-cell sets used in this work were obtained from public open resources, as shown in Table 1. The most relevant data generated during the development of this work are included in the Supplementary Material. All other data supporting the results of this study are available upon request from the corresponding author: Javier De Las Rivas (jrivas@usal.es).

## Supplementary Materials

The following supporting information can be downloaded at: https://www.mdpi.com/article/10.3390/ijms26020805/s1.
