# Supplementary figures and images for: Novel Assignment of Gene Markers to Hematological and Immune Cells Based on Single-Cell Transcriptomics
Source: Int J Mol Sci. 2025 Jan 18;26(2):805. doi: 10.3390/ijms26020805 (PMC11765818; doi:10.3390/ijms26020805)

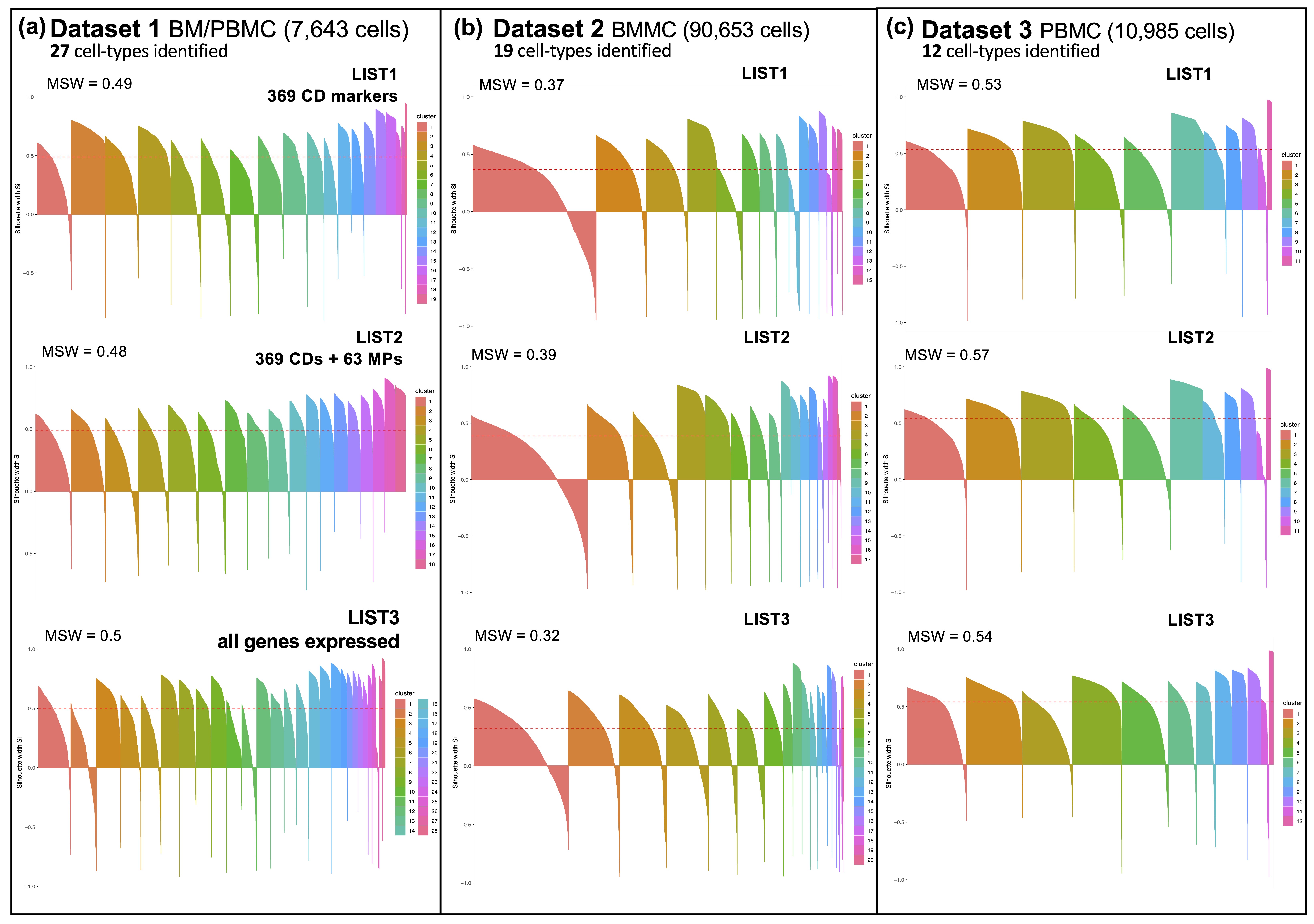

Supplement: Supplementary file 1 [file ijms-26-00805-s001.zip › Supplementary-Figure_S1.jpg]
